# Cancers attributable to diet in Italy

**DOI:** 10.1002/ijc.35227

**Published:** 2024-10-24

**Authors:** Federica Turati, Gianfranco Alicandro, Giulia Collatuzzo, Claudio Pelucchi, Matteo Malvezzi, Fabio Parazzini, Eva Negri, Paolo Boffetta, Carlo La Vecchia, Matteo Di Maso

**Affiliations:** ^1^ Department of Clinical Sciences and Community Health, Department of Excellence 2023–2027, Branch of Medical Statistics, Biometry and Epidemiology “G.A. Maccacaro” University of Milan Milan Italy; ^2^ Department of Pathophysiology and Transplantation Università degli Studi di Milano Milan Italy; ^3^ Cystic Fibrosis Centre Fondazione IRCCS Ca' Granda Ospedale Maggiore Policlinico Milan Italy; ^4^ Department of Medical and Surgical Sciences University of Bologna Bologna Italy; ^5^ Department of Medicine and Surgery University of Parma Parma Italy; ^6^ Department of Clinical Sciences and Community Health, Department of Excellence 2023–2027 University of Milan Milan Italy; ^7^ Stony Brook Cancer Center Stony Brook University Stony Brook New York USA; ^8^ Department of Family, Population and Preventive Medicine, Renaissance School of Medicine Stony Brook University Stony Brook New York USA

**Keywords:** attributable cancer cases, attributable cancer deaths, dietary factors, population attributable fraction

## Abstract

Cancer burden can be reduced by controlling modifiable risk factors, including diet. We provided an evidence‐based assessment of cancer cases and deaths attributable to diet in Italy in 2020. We considered dietary factor‐cancer type pairs for which the World Cancer Research Fund/American Institute for Cancer Research – Continuous Update Project reported either ‘convincing’ or ‘probable’ evidence of causal association. Relative risks were retrieved from recent meta‐analyses and dietary intakes (around 2005) from a national food consumption survey. Sex‐specific population attributable fractions (PAFs) were computed by comparing the distribution of dietary intakes in the Italian population against counterfactual scenarios based on dietary recommendations. Using data from national cancer and mortality registries in 2020, we estimated the number of attributable cancer cases and deaths, assuming ~15‐year lag period. Unhealthy diet accounted for 6.3% (95% CI: 2.5%–9.9%) of all cancer cases in men and 4.5% (95% CI: 1.7%–7.4%) in women. PAFs of colorectal cancer were 10.5% and 7.0% for any intake of processed meat, 3.3% and 2.0% for high red meat, 4.8% and 4.3% for low dairy products, and 7.9% and 9.0% for low fiber intakes in men and women, respectively. PAFs for low intake of non‐starchy vegetables and fruit ranged from 0.8% to 16.5% in men and 0.6%–17.8% in women for cancers of the aerodigestive tract. The estimated cancer burden associated with unfavorable dietary habits in Italy is considerable, but appears lower than for other high‐income countries, reflecting the typically Mediterranean diet.

## Graphical abstract

What's new?

The Italian diet is primarily Mediterranean, being based on plant foods and on relatively low intake of red and processed meat. The impact of this diet on cancer burden, however, remains uncertain. Here, the authors examined cancer cases and deaths linked to diet in the Italian population. Analyses indicate that 6.3 percent of cancers in men and 4.5 percent in women can be attributed to unhealthy diet. Risk was highest for colorectal cancer. Relative to other high‐income countries, Italy has a lower estimated cancer burden attributable to unhealthy diet. The findings may inform interventions to reduce diet‐related cancer burden.

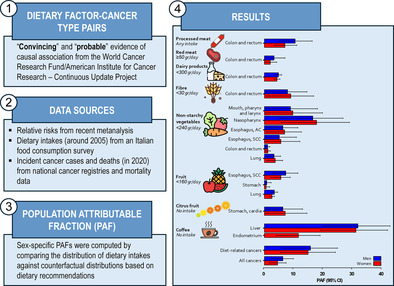

## 1 INTRODUCTION

Lifestyle and environmental factors play a major role on cancer risk, offering important avenues for prevention.^1^, ^2^, ^3^ In particular, a large body of epidemiological evidence indicates poor diet as an amenable risk factor for cancer.^4^ Several studies reported a direct relationship between high processed and red meat intakes and digestive tract cancers, especially colorectal cancer.^5^, ^6^, ^7^, ^8^ An inverse association was reported for dairy products and colorectal cancer as well.^8^, ^9^ Multiple studies found reduced risks of cancers mainly of the upper aerodigestive tract for diets rich in vegetables and fruit, and their related compounds (e.g., fibers and antioxidants).^5^, ^10^

The population attributable fraction (PAF) is an epidemiological measure which quantifies the proportion of cases that would have not occurred in the absence of the exposure. It depends on both the strength of the association between the exposure and the disease and the prevalence of the exposure.^11^ PAF estimates of cancer due to diet have been calculated for various countries, including France,^12^ Switzerland,^13^ Denmark,^14^ UK,^15^, ^16^, ^17^, ^18^ Brazil,^19^ China,^20^, ^21^, ^22^, ^23^ Japan,^24^, ^25^ Korea,^26^ the US,^27^, ^28^ Canada,^29^, ^30^, ^31^ and Australia.^32^, ^33^ A systematic contemporary assessment for Italy is, however, lacking. Quantifying the contribution of unhealthy dietary habits to the cancer burden at the country level is crucial to guide public health recommendations aiming at improving diet and, in turn, population's health.

The aim of our study is to conduct an evidence‐based evaluation of the PAF of cancer due to a poor diet in the Italian population. The Italian diet, which is typically Mediterranean, based on plant foods and characterized by relatively low amounts of red and processed meat, makes this assessment of particular interest.

## 2 METHODS

Within the framework of a global analysis of the cancer burden attributable to modifiable risk factors in Italy,^34^, ^35^, ^36^, ^37^, ^38^ we estimated here PAFs due to unhealthy dietary habits. We selected dietary factors and the corresponding related cancer sites (Table 1) for which the World Cancer Research Fund/American Institute for Cancer Research – Continuous Update Project (WCRF/AICR‐CUP) reported ‘convincing’ (i.e., processed meat for colorectal cancer)^8^ or ‘probable’ evidence (i.e., red meat, dairy products, and fiber for colorectal cancer; citrus fruit for cardia stomach cancer; coffee for liver and endometrial cancers)^5^, ^8^, ^10^, ^39^ for a causal association. We did not consider aflatoxin, arsenic in water, and high‐dose beta carotene supplements among factors with a ‘convincing’ association, and salt‐preserved foods, cantonese‐style salted fish, mate, fast foods, and calcium supplements among those with a ‘probable’ association because these dietary factors are not relevant exposures in our population, or we could not identify reliable estimates of intake. In addition, we did not consider wholegrains for colorectal cancer (‘probable’ association) since we included fiber, which strongly correlate with the former, and no adequate data on wholegrain intake for the Italian population were available. Likewise, no data on glycemic load (‘probable’ association with endometrial cancer) were available, and therefore we did not include it in our analysis. The WCRF/AICR judged as ‘probable’ the association between non‐starchy vegetables and fruit and aerodigestive tract cancers, considering that, although the evidence for single cancer sites is ‘limited‐suggestive’, the pattern of association is consistent and in the same direction.^10^ We considered cancer sites as follows: mouth, pharynx, larynx, nasopharynx, esophagus (adenocarcinoma and squamous cell carcinoma), colorectum and lung for non‐starchy vegetables; and esophagus (squamous cell carcinoma), stomach and lung for fruit; since available epidemiological evidence for vegetable/fruit‐cancer associations has focused mainly on single cancer sites and considered separately the effect of vegetables and fruit, as reported in the WCRF/AICR‐CUP.^10^

**TABLE 1:** Relative risk (RR) and corresponding 95% confidence interval (CI) used for each dietary factor‐cancer type pair.

Dietary factor	Cancer type	RR (95% CI), increase in amount	References
‘Convincing’ association^a^			
Processed meat	Colon and rectum	1.16 (1.10–1.28), 50 g/day	Viera et al.^40^
‘Probable’ association^a^			
Red meat	Colon and rectum	1.12 (1.00–1.25), 100 g/day	Viera et al.^40^
Dairy products	Colon and rectum	0.87 (0.83–0.90), 400 g/day	Viera et al.^40^
Fiber	Colon and rectum	0.93 (0.87–1.00), 10 g/day	WCRF/AIRC CUP, colorectal cancer^8^
Non‐starchy vegetables	Mouth, pharynx, and larynx	0.96 (0.92–1.01), 25 g/day	Maasland et al.^43^
	Nasopharynx	0.74 (0.60–0.92), 100 g/day	Unpublished data
	Esophagus, AC	0.89 (0.80–0.99), 100 g/day	Vingeliene et al.^41^
	Esophagus, SCC	0.91 (0.81–1.03), 100 g/day	Vingeliene et al.^41^
	Colon and rectum	0.98 (0.96–0.99), 100 g/day	Viera et al.^40^
	Lung	0.94 (0.89–0.98)^b^, 100 g/day	Viera et al.^42^
Fruit	Esophagus, SCC	0.84 (0.75–0.94), 100 g/day	Vingeliene et al.^41^
	Stomach	0.98 (0.94–1.02), 100 g/day	WCRF/AIRC CUP, stomach cancer^8^
	Lung	0.92 (0.89–0.95)^b^, 100 g/day	Viera et al.^42^
Citrus fruit	Stomach, cardia	0.87 (0.76–0.99), any versus no intake	Vingeliene et al.^44^
Coffee	Liver	0.66 (0.55–0.78), any versus no intake	Bravi et al.^45^
	Endometrium	0.87 (0.79–0.95), any versus no intake	Crous‐Bou et al.^46^

Abbreviations: AC, adenocarcinoma; SCC, squamous cell carcinoma.

^a^ Defined according to the World Cancer Research Fund/American Institute for Cancer Research (WCRF/AICR).^5^, ^8^, ^10^, ^39^

^b^ WCRF/AICR evidence on non‐starchy vegetables or fruit intake is restricted to former and current smokers. Data on non‐starchy vegetables or fruit intake in Italy are available for smokers and non‐smokers combined. We extracted RRs of lung cancer for non‐starchy vegetables and fruit intake among smokers and non‐smokers combined.

### 2.1 RR data

The RRs for the association between the increase in amount of intakes (expressed in g/day) and the corresponding cancer types were obtained from meta‐analyses and pooled analyses^8^, ^40^, ^41^, ^42^ and, in one case, from a cohort study.^43^ For citrus fruit and coffee, we extracted the RRs for any versus no intake from metanalyses.^44^, ^45^, ^46^ Original data from an Italian case–control study^47^ were used to estimate the odds ratio between non‐starchy vegetables and nasopharyngeal cancer risk by means of a logistic regression model adjusted for area of residence, sex, age, education, smoking, and total energy intake. The RRs for each pairwise association are reported in Table 1.

### 2.2 Dietary intake data

Dietary intake data were obtained from the Italian National Food Consumption Survey INRAN‐SCAI 2005–06, conducted between 2005 and 2006 on 3323 subjects belonging to 1329 households using consecutive 3‐day food records.^48^, ^49^, ^50^, ^51^ For each dietary factor, we extracted the proportion of non‐consumers and consumers among subjects aged 18–65 years (men = 1068 and women = 1244), and for consumers, we extracted the mean and standard deviation of intake (Table 2).

**TABLE 2:** Distribution of dietary intakes by sex in Italy in 2005–2006^a^.

Dietary factor	Men		Women	
	Prevalence of consumers (%)	Mean (SD) intake among consumers (g/day)	Prevalence of consumers (%)	Mean (SD) intake among consumers (g/day)
Processed meat	87	40.7 (31.0)	80	29.3 (22.4)
Red meat	83	75.0 (51.4)	80	60.3 (37.8)
Fiber	100	19.6 (7.3)	100	17.7 (6.3)
Dairy products	99	178.5 (126.0)	99	194.5 (117.0)
Non‐starchy vegetables	~100	232.9 (120.2)	~100	213.3 (102.6)
Fruit	93	214.7 (157.8)	95	228.2 (143.8)
Citrus fruit	45		51	
Coffee	91		88	

^a^ Extracted from the Italian National Food Consumption Survey INRAN‐SCAI 2005–06.^48^, ^49^, ^50^, ^51^

In consumers, we estimated the intake distribution for each dietary factor by means of a gamma distribution^52^ with shape and scale parameters based on the observed mean and standard deviation of intake (i.e., by the INRAN‐SCAI 2005‐06). The gamma distribution is a flexible two‐parameter distribution that allows to fit a variety of shapes, including both symmetric and highly skewed ones. We used the method of moments to estimate gamma parameters, assuming means and standard deviations from the INRAN‐SCAI 2005‐06 as population means and standard deviations of dietary intakes. The overall distribution of dietary intake for men and women separately was obtained by combining the prevalence of non‐consumers with the gamma‐derived intervals of intakes among consumers. In particular, we considered 11 exposure categories, i.e., no intake category and 10 categories of intake with the same frequency of consumers according to the estimated gamma quantiles (Figures S1 and S2).

### 2.3 Recommended intake

PAFs were calculated against counterfactual scenarios based on recommended dietary intakes by the WCRF/AICR or other Institutions (Table 3).^53^, ^54^, ^55^ Specifically, the WCRF/AICR recommends consuming very little, if any, processed meat; no more than about three portions/week of red meat (i.e., <~350–500 g/week); and at least 30 g/day of fibre.^53^ Thus, our counterfactual scenarios were as follows: 0 g/day of processed meat, <50 g/day of red meat (lower cut‐off of the recommended intake), and ≥30 g/day of fiber. The WCRF/AICR, the Food and Agriculture Organization of the United Nations (FAO) and the World Health Organization (WHO) recommend the intake of at least five servings/day of fruit and vegetables (i.e., ≥400 g/day; one serving ~80 g).^53^, ^54^, ^55^ We set counterfactual scenarios separately for non‐starchy vegetables and fruit of, respectively, ≥240 g/day and ≥160 g/day based on most country‐specific guidelines suggesting at least three servings/day should come from vegetables.^56^ No WCRF/AICR recommendation exists for dairy products; we set the corresponding counterfactual scenario as ≥300 g/day since more than half of European dietary guidelines recommend 2–4 servings of dairy products per day (i.e., ~300–450 g/day).^57^ No recommendation is provided for citrus fruit and coffee, and we considered any intake as counterfactual scenario.

**TABLE 3:** Population attributable fraction (PAF), observed and attributable cancer cases in 2020, and corresponding 95% confidence interval (CI) for each dietary factor‐cancer type pair by sex in Italy.

Dietary factor/cancer type	Counterfactual distribution of dietary factor^a^	Men			Women		
		PAF (95% CI), %	Observed cases, *n*	Attributable cases (95% CI), *n*	PAF (95% CI), %	Observed cases, *n*	Attributable cases (95% CI), *n*
‘Convincing’ association^b^							
Processed meat	0 g/day						
Colon and rectum [C18–C20]		10.5 (5.0–16.3)	23,420	2459 (1171–3817)	7.0 (3.4–11.0)	20,282	1420 (690–2231)
‘Probable’ association^b^							
Red meat	Intake of <50 g/day						
Colon and rectum [C18–C20]		3.3 (0.1–7.0)	23,420	773 (23–1639)	2.0 (0.1–4.2)	20,282	406 (20–852)
Dairy products	Intake of ≥300 g/day						
Colon and rectum [C18–C20]		4.8 (3.6–5.9)	23,420	1124 (843–1382)	4.3 (3.2–5.3)	20,282	872 (649–1075)
Fiber	Intake of ≥30 g/day						
Colon and rectum [C18–C20]		7.9 (0.4–14.5)	23,420	1850 (94–3396)	9.0 (0.5–16.8)	20,282	1825 (101–3407)
Non‐starchy vegetables	Intake of ≥240 g/day						
Mouth [C00‐–C10; C12–C13], pharynx [C14], and larynx [C32]		8.9 (0.0–18.1)	6855	610 (0–1241)	9.7 (0.0–19.8)	2462	239 (0–487)
Nasopharynx [C11]		16.5 (5.2–26.8)	421^c^	69 (22–113)	17.8 (5.7–29.0)	118^c^	21 (7–34)
Esophagus, AC [C15]		6.3 (0.7–11.4)	564^d^	36 (4–64)	6.9 (0.7–12.6)	226^d^	16 (2–28)
Esophagus, SCC [C15]		5.1 (0.0–10.9)	1146^d^	58 (0–125)	5.6 (0.0–12.1)	458^d^	26 (0–55)
Colon and rectum [C18–C20]		1.1 (0.3–1.8)	23,420	258 (70–422)	1.2 (0.3–2.0)	20,282	243 (61–406)
Lung [C34]		3.3 (0.8–5.5)	27,554	909 (220–1515)	3.7 (0.9–6.2)	13,328	493 (120–826)
Fruit	Intake of ≥160 g/day						
Esophagus, SCC [C15]		7.3 (2.8–11.4)	1146^d^	84 (32–131)	5.6 (2.1–8.8)	458^d^	26 (10–40)
Stomach [C16]		0.8 (0.0–2.3)	8458	68 (0–195)	0.6 (0.0–1.8)	6098	37 (0–110)
Lung [C34]		3.4 (2.3–4.5)	27,554	937 (634–1240)	2.6 (1.7–3.4)	13,328	347 (227–453)
Citrus fruit	Any intake						
Stomach, cardia [C16.0]		6.3 (0.4–13.0)	1269	80 (5–165)	7.1 (0.4–14.5)	915	65 (4–133)
Coffee	Any intake						
Liver [C22]		31.9 (19.7–42.8)	8978	2864 (1769–3843)	31.2 (19.2–42.0)	4034	1259 (775–1694)
Endometrium [C54.1]		–	–	–	11.6 (4.1–19.0)	8335	967 (342–1584)
Diet‐related cancers^e^		15.7 (6.3–24.9)	77,396	12,179 (4887–19,288)	14.9 (5.4–24.2)	55,341	8262 (3008–13,415)
All cancers^f^		6.3 (2.5–9.9)	194,754	12,179 (4887–19,288)	4.5 (1.7–7.4)	181,857	8262 (3008–13,415)

^a^ Defined according to dietary intake recommendations of the World Cancer Research Fund/American Institute for Cancer Research (WCRF/AICR) or other Institutions.^53^, ^54^, ^55^

^b^ Defined according to the World Cancer Research Fund/American Institute for Cancer Research (WCRF/AICR).^5^, ^8^, ^10^, ^39^

^c^ Nasopharyngeal cancer cases were estimated as 8.7% and 6.9% of the total oral and pharyngeal cancers in men (*n* = 4839) and women (*n* = 1716), respectively, according to a previously defined algorithm.^34^

^d^ Adenocarcinomas and squamous cell carcinomas of the esophagus were estimated as 33% and 67% of the total esophageal cancers, respectively, according to a previously defined algorithm.^34^

^e^ Diet‐related cancers included the following cancer sites: mouth, pharynx, larynx, nasopharynx, esophagus (adenocarcinoma and squamous cell carcinoma), stomach, colorectum, liver, lung, and endometrium.

^f^ All cancers excluding non‐melanoma skin cancer.

### 2.4 Population attributable fractions

For each pair of dietary factor and cancer type, sex‐specific PAFs were estimated by comparing the distributions of dietary intakes in the Italian population against the corresponding counterfactual distributions (recommended intakes) using the same methodology as described in Parkin and Boyd 2011.^16^ Specifically, PAFs were calculated by:

$$\mathrm{PAF}=\frac{\sum_{k=1}^{K}\left({\mathrm{ERR}}_{k}\cdot {\mathrm{Pr}}_{k}\right)}{1+\sum_{k=1}^{K}\left({\mathrm{ERR}}_{k}\cdot {\mathrm{Pr}}_{k}\right)}$$

where ${\mathrm{Pr}}_{k}$ is the proportion of subjects in the k‐th intake category, and ${\mathrm{ERR}}_{k}$ is the excess RR in the k‐th intake category expressed by:

$${\mathrm{ERR}}_{k}=\left\{\exp\left[\ln\left({\mathrm{RR}}_{1g}\right)\cdot {\mathrm{Dev}}_{k}\right]-1\right\}$$

where $\ln\left({\mathrm{RR}}_{1g}\right)$ is the natural logarithm of the RR for an increment of 1 g/day in dietary factor intake and ${\mathrm{Dev}}_{k}$ is the deviation (excess or deficit) from the counterfactual scenario in the k‐th intake category. We defined ${\mathrm{Dev}}_{k}$ as the difference between the midpoint of each intake category and the recommended intake. For protective dietary factors, we used the reciprocal of the RR and therefore the $\ln\left(1/{\mathrm{RR}}_{1g}\right)$ represents the increase in risk (log‐scale) for a deficit of 1 g/day in dietary factor intake.

According to the counterfactual distribution considered for citrus fruit and coffee consumption, the above formulation of PAF collapses to the Levin's formula^58^ for a dichotomous exposure:

$$\mathrm{PAF}=\frac{\left(\mathrm{RR}-1\right)\cdot \mathrm{Pr}}{1+\left[\left(\mathrm{RR}-1\right)\cdot \mathrm{Pr}\right]}$$

where RR is the relative risk for consumption versus no consumption and Pr is the proportion of consumers.

To compute the 95% confidence intervals (CIs) for PAFs, we performed 10,000 Monte Carlo simulations^59^ considering both the variability of RRs and dietary factors. In particular, we simulated asymptotic distributions for RRs and each dietary factors considering a normal distribution for the logarithm of the RR; a normal distribution for the mean intake of continuous dietary factors (in order to generate gamma distributions with simulated means and the observed standard deviation); and the normal approximation of the binomial distribution for the proportion of consumers for dichotomous dietary factors. For each simulation, we calculated PAFs and used the 2.5th and 97.5th quantiles of the PAF distribution as the approximate 95% CI. When the lower bound of 95% CI was negative, we set it to zero.

### 2.5 Cancer data and attributable cancers

Attributable cancer cases and deaths were estimated by multiplying sex‐ and cancer‐specific PAFs by the number of incident cases and deaths occurred in Italy in 2020, respectively (ICD‐10 codes are reported in Table S1). The estimated number of cancer diagnoses in 2020 (projected data on diagnoses registered until 2016) was extracted from the Italian Association of Cancer Registries^60^; the number of cancer deaths that occurred in 2020 was extracted from the national vital statistics on causes of death.^61^ From these sources, data on cancer cases and deaths were not available for some cancer subtypes and, therefore, these were estimated as follows^34^: cardia gastric cancers (cases and deaths) were estimated as 33% of all gastric cancers; adenocarcinomas and squamous cell carcinomas of the esophagus (cases and deaths) as, respectively, 33% and 67% of all esophageal cancers; and nasopharyngeal cancers (cases and deaths) as 8.7% of oral and pharyngeal cancers.

Using dietary data in 2005–2006 and cancer data in 2020 we accounted for a ~15‐year lag period between dietary exposure and cancer diagnosis/death. The 95% CIs for attributable cases and deaths were calculated by using the 95% CI limits of PAFs. All analyses were conducted using R version 4.3.1 (R Core Team, 2023).

## 3 RESULTS

Table 3 reports sex‐specific PAFs and attributable cases with the corresponding 95% CIs. Overall, the dietary factors considered in this study were responsible for 12,179 (95% CI: 4887–19,288) incident cancer cases among men in 2020 in Italy, corresponding to a PAF of all cancers of 6.3% (95% CI: 2.5%–9.9%). The fraction of colorectal cancers attributable to processed meat intake (the only ‘convincing’ association reported by the WCRF/AICR) was 10.5% (95% CI: 5.0%–16.3%) with a burden of cases of 2459 (95% CI: 1171–3817). Among the ‘probable’ associations suggested by WCRF/AICR, the fraction of colorectal cancer cases attributable to high intake of red meat and low intake of dairy products were 3.3% (95% CI: 0.1%–7.0%) and 4.8% (95% CI: 3.6%–5.9%), respectively. Low fiber intake accounted for 7.9% (95% CI: 0.4%–14.5%) of colorectal cancer cases. PAFs for low non‐starchy vegetable consumption ranged from 1.1% (95% CI: 0.3%–1.8%) for colorectal cancer to 16.5% (95% CI: 5.2%–26.8%) for nasopharyngeal cancer. Low fruit intake was responsible for 7.3% (95% CI: 2.8%–11.4%) of squamous cell carcinomas of the esophagus, 3.4% (95% CI: 2.3%–4.5%) of lung cancers, and 0.8% (95% CI: 0.0%–2.3%) of gastric cancers. No consumption of citrus fruit resulted in a fraction of cardia gastric cancers of 6.3% (95% CI: 0.4%–13.0%), and no coffee consumption in a fraction of liver cancers of 31.9% (95% CI: 19.7%–42.8%).

Among women, a poor diet accounted for 8262 (95% CI: 3008–13,415) diagnoses of all cancers in 2020 in Italy corresponding to a PAF of 4.5% (95% CI: 1.7%–7.4%). The share of colorectal cancers attributable to processed meat was 7.0% (95% CI: 3.4%–11.0%); in absolute numbers, it translated into 1420 (95% CI: 690–2231) cases. Regarding the ‘probable’ associations, PAFs for colorectal cancer due to high red meat and low dairy product intakes were 2.0% (95% CI: 0.1%–4.2%) and 4.3% (95% CI: 3.2%–5.3%), respectively. The fraction of colorectal cancer cases attributable to low fiber intake was 9.0% (95% CI: 0.5%–16.8%). PAFs for low intake of non‐starchy vegetables ranged from 1.2% (95% CI: 0.3%–2.0%) for colorectal cancer to 17.8% (95% CI: 5.7%–29.0%) for nasopharyngeal cancer. Low fruit consumption was responsible for 5.6% (95% CI: 2.1%–8.8%) of squamous cell carcinomas of the esophagus, 2.6% (95% CI: 1.7%–3.4%) of lung cancer cases, and 0.6% (95% CI: 0.0%–1.8%) of gastric cancer cases. The share of cardia stomach cancer cases due to no consumption of citrus fruit was 7.1% (95% CI: 0.4%–14.5%). The lack of coffee consumption accounted for 31.2% (95% CI: 19.2%–42.0%) and 11.6% (95% CI: 4.1%–19.0%) of liver and endometrial cancer cases, respectively.

Attributable cancer deaths are reported in Table S2. A poor diet was associated with over 10,000 cancer deaths, i.e., 6674 (95% CI: 2815–10,412) in men and 4074 (95% CI: 1573–6509) in women.

## 4 DISCUSSION

The present study provides a comprehensive assessment of the fraction and number of cancer cases and deaths attributable to diet in the Italian population. The dietary factors considered in our analysis were responsible for 6.3% and 4.5% of all cancer cases in Italy in 2020 among men and women, respectively. For cancer‐specific PAFs, no major differences were observed according to sex, reflecting similar dietary intakes in men and women. Specifically, PAFs of colorectal cancer due to processed meat intake were 10.5% for men and 7.0% for women. Among other dietary factors considered, PAFs of colorectal cancer were 3.3% (men) and 2.0% (women) for high red meat intake; 4.8% (men) and 4.3% (women) for low intake of dairy products; and 7.9% (men) and 9.0% (women) for low fiber intake. The fractions of cases due to low non‐starchy vegetables and fruit intake ranged from less than 1% for colorectal cancer to approximately 17% for nasopharyngeal cancer in both sexes.

Italian dietary habits at least in part reflect the Mediterranean diet, characterized by a low intake of processed and red meat; a moderate intake of dairy products (mostly cheese and yogurt), poultry and fish; high intake of vegetables, fresh fruits, legumes, bread and other cereals (generally minimally refined), potatoes, and nuts; and a high intake of olive oil (especially virgin and extra‐virgin) used as the main source of fats.^62^ The Mediterranean diet has been extensively associated with a portfolio of health benefits including a reduced risk of developing several cancer types, as well as a lower cancer‐specific and overall mortality.^63^, ^64^, ^65^ Consistent with Italian dietary habits, PAFs for cancer due to dietary factors in this population are expected to be lower than those among populations with less healthy dietary patterns. However, PAF depends not only on the population's dietary habits but also on the choice of the RRs and counterfactual scenarios, making results across studies not directly comparable. In addition, different tools used to estimate population dietary intakes (e.g., 24‐h dietary recall, food frequency questionnaires, and food records) may further hamper the comparison of results across studies.

Nearly all previous studies^12^, ^14^, ^18^, ^19^, ^23^, ^25^, ^27^, ^29^, ^31^, ^66^ assessing the PAF of colorectal cancer for processed meat intake used an analytical approach similar to the one used in this study, including similar RRs (from 1.11 to 1.24 for an intake of 50 g/day) and counterfactual scenarios (i.e., 0 g/day for the majority of the studies). Therefore, the higher PAFs observed in France (12.7% for men and 8.8% for women),^12^ Switzerland (13.8% for men and 8.1% for women),^13^ UK (16.1% for men and 8.6% for women),^18^ and Denmark (15.8% for men and 8.0% for women)^14^ largely reflect higher processed meat intakes in these populations (mean intakes ranged from 43.9 to 74.0 g/day for men and from 29.5 to 37.0 g/day for women) than the Italian one (40.7 g/day and 29.3 g/day for men and women, respectively). Likewise, the lower PAFs reported in Brazil (6.6% for men),^19^ Canada (from 3.8% to 4.9% for men; from 1.6% to 3.4% for women),^29^, ^31^ Eastern Mediterranean Region Countries (3.1% for men and 3.0% for women),^66^ Japan (4.3% for men and 3.3% for women),^25^ and China (1.0% for men and 0.9% for women)^23^ derived mainly from a lower processed meat consumption in these Countries, with mean intakes of <19.5 g/day for men and <10.5 g/day for women.

Compared to our study, similar fractions of colorectal cancer cases attributable to red meat were observed for populations in Eastern Mediterranean Region (2.2% for men and 2.1% for women),^66^ likely explained by comparable dietary intakes in these countries (mean intake of 55.9 g/day for men and women combined) and Italy. Studies conducted in the US (6.6% for men and 3.9% for women),^27^ Canada (5.8% for men and 4.6% for women),^31^ France (5.8% for men and 3.3% for women),^12^ and Switzerland (5.2% for men)^13^ reported higher PAFs for red meat intake than our estimates. Different counterfactual scenarios, RRs, and intake levels all likely contributed to such differences. Regarding dairy products, the higher consumption in France resulted in lower estimates of colorectal cancer cases avoidable by optimal consumption (2.1% for men and 2.7% for women)^12^ compared to those in Italy.

As for fiber intake, previous investigations relied on intake levels (mean intake ~18–24 g/day for men and ~15–20 g/day for women) and counterfactuals (≥30 g/day in the majority of the studies) similar to those used here. Thus, the large variability in PAFs (from 5.7% in France^12^ to 24.6% in UK^18^ for men and from 7.1% in Japan^67^ to 31.0% in UK^18^ for women) was mainly due to the different RR estimates used. Similarly, PAFs for inadequate fruit and vegetable consumption were heterogeneous across populations. For most cancer types included in the present analysis (i.e., mouth, pharynx, and larynx; nasopharynx; squamous cell carcinoma of the esophagus; colon and rectum; lung; and stomach), our PAFs were lower than those of other studies.^12^, ^13^, ^17^, ^20^, ^21^, ^22^, ^23^, ^27^, ^30^, ^32^, ^66^, ^68^ This may be largely ascribable to the higher intake of fruit and vegetables in Italy; still, the choice of RRs and counterfactual scenarios may have played a role.

Few studies^13^, ^69^, ^70^ quantified the burden of liver and endometrial cancer cases due to coffee consumption. For both cancer types, our PAFs were higher than those reported in the US,^69^, ^70^ due to the different prevalence of coffee consumers and RRs used. A Swiss study^13^ showed PAF estimates of liver and endometrial cancers similar to those presented here, reflecting a similar pattern of coffee consumption.

Primary prevention by controlling modifiable risk factors is the best option to reduce cancer burden. Although strategies for reducing smoking, alcohol, overweight, and cancer‐related infections remain the cornerstone of cancer prevention, according to our findings the adoption of healthy dietary habits would result in a considerable fraction of avoidable cancer cases in Italy (6.3% in men and 4.5% in women). We considered dietary factors and corresponding cancer types according to the updated guidelines of the WCRF/AICR. Other studies, however, considered different sets of dietary factor‐cancer type pairs suggesting major uncertainties about the causal relationship between diet and cancer risk.

Among study's strengths, we used nationally representative data for dietary intakes, large meta‐analyses and pooled analyses for RRs, and common methodologies for estimating PAFs and corresponding 95% CIs. We also accounted for a plausible latency period (i.e., ~15‐year) between dietary intakes (i.e., around 2005) and cancer diagnosis (i.e., 2020), and provided a measure of variability of our PAF estimates.

In the national survey used as data source, dietary intakes were self‐recorded potentially leading to inaccurate measurement of food consumption. In addition, our estimated intakes deriving from a probability distribution may not be accurate for all dietary factors considered. However, the gamma distribution was used in several previous studies and in similar contexts.^28^, ^71^, ^72^ Furthermore, our PAF estimates are consistent with those from other studies which do not use a probability distribution for dietary intakes. In addition, it has been argued that in case of confounding in the relationship between risk factor and disease, the Levin's formula is asymptotically biased and Miettinen's formula is more accurate.^73^ Nevertheless, Miettinen's formula requires prevalence data among cases that were not available. As in most prior studies on PAF, we applied the Levin's formula plugging‐in adjusted RRs. However, residual confounding by lifestyle, if any, would have led to overestimation of PAFs. Most RR estimates were derived from studies on cancer incidence. Despite this, we used the same RRs for incidence and mortality, thus assuming no effect of diet on cancer survival, possibly introducing bias in estimated attributable deaths. In addition, cancer mortality in 2020 may be affected by COVID‐19 pandemic; however, no relevant temporal difference emerged comparing national cancer mortality data in the years around 2020.^61^, ^74^ This issue is not relevant for incidence data, since we extracted cancer cases estimated to have occurred in 2020 based on available registered cancer data until 2016. Our estimates provide fractions of cancer cases/deaths attributable to dietary factors acting in isolation, without considering potential interactions among dietary factors. We could not consider interactions in our calculation, as no national information on the combined intakes of multiple dietary factors was available. Likewise, we could not account for the possible role of the substitution effect, for instance, of increasing vegetables and fruit at the expense of red meat intake. Recently, an approach based on pathway‐specific PAFs has been proposed^75^ to address this issue, but it requires individual‐level data. Furthermore, we could not compute age‐specific PAFs because of missing data on dietary intake in strata of age. Lastly, the ‘overall’ estimates may be overestimated since they resulted from summing up the contributions of the individual dietary factors, ignoring their possible overlapping effects on the same cancer site. Alternatively, the overall impact of diet on cancer burden could be greater than estimated since other dietary aspects (e.g., salted‐preserved foods, fast foods, and foods with high glycemic index) may influence the risk of selected cancers as well.

## 5 CONCLUSIONS

This analysis offers robust data to inform interventions aimed at reducing the burden of cancer related to dietary factors in Italy. We found that unfavorable dietary habits cause a considerable proportion of cancers in the country. Despite challenges in comparisons across studies, PAFs due to poor diet in Italy appear lower than for other high‐income countries, likely reflecting the healthier (Mediterranean) dietary pattern of the Italian population.

## AUTHOR CONTRIBUTIONS

**Federica Turati:** Data curation; formal analysis; investigation; methodology; visualization; writing – original draft. **Gianfranco Alicandro:** Investigation; writing – review and editing. **Giulia Collatuzzo:** Conceptualization; investigation; writing – review and editing. **Claudio Pelucchi:** Investigation; writing – review and editing. **Matteo Malvezzi:** Investigation; writing – review and editing. **Fabio Parazzini:** Investigation; writing – review and editing. **Eva Negri:** Funding acquisition; investigation; supervision; writing – review and editing. **Paolo Boffetta:** Conceptualization; investigation; supervision; writing – review and editing. **Carlo La Vecchia:** Investigation; supervision; writing – review and editing. **Matteo Di Maso:** Data curation; formal analysis; investigation; methodology; visualization; writing – original draft.

## FUNDING INFORMATION

This work was conducted with the contribution of the Fondazione AIRC per la Ricerca sul Cancro (IG grant N. 22987).

## CONFLICT OF INTEREST STATEMENT

The authors have no potential conflicts of interest to declare.

## Supporting information


Data S1.

## Data Availability

All supporting data and source code are publicly available on Github (https://github.com/matte0dimaso/PAF_Diet_Italy). Further information is available from the corresponding author upon request.

## References

[1] Collaborators GBDCRF . The global burden of cancer attributable to risk factors, 2010–19: a systematic analysis for the global burden of disease study 2019. Lancet. 2022;400(10352):563‐591. doi:10.1016/S0140-6736(22)01438-6 35988567 PMC9395583

[2] Lichtenstein P , Holm NV , Verkasalo PK , et al. Environmental and heritable factors in the causation of cancer‐analyses of cohorts of twins from Sweden, Denmark, and Finland. N Engl J Med. 2000;343(2):78‐85. doi:10.1056/NEJM200007133430201 10891514

[3] Blot WJ , Tarone RE . Doll and Peto's quantitative estimates of cancer risks: holding generally true for 35 years. J Natl Cancer Inst. 2015;107(4):djv044. doi:10.1093/jnci/djv044 25739419

[4] McCullough ML , Giovannucci EL . Diet and cancer prevention. Oncogene. 2004;23(38):6349‐6364. doi:10.1038/sj.onc.1207716 15322510

[5] World Cancer Research Fund/American Institute for Cancer Research . Diet, nutrition, physical activity and cancer: a global perspective. Continuous update project expert report 2018. https://www.wcrf.org/wp-content/uploads/2021/02/Summary-of-Third-Expert-Report-2018.pdf

[6] International Agency for Research on Cancer . Red meat and processed meat. IARC monographs on the evaluation of carcinogenic risks to humans. Vol 114. International Agency for Research on Cancer; 2018.

[7] Di Y , Ding L , Gao L , Huang H . Association of meat consumption with the risk of gastrointestinal cancers: a systematic review and meta‐analysis. BMC Cancer. 2023;23(1):782. doi:10.1186/s12885-023-11218-1 37612616 PMC10463360

[8] World Cancer Research Fund/American Institute for Cancer Research . Continuous update project expert report 2018. Diet, nutrition, physical activity and colorectal cancer. https://www.wcrf.org/wp-content/uploads/2021/02/Colorectal-cancer-report.pdf

[9] Papadimitriou N , Markozannes G , Kanellopoulou A , et al. An umbrella review of the evidence associating diet and cancer risk at 11 anatomical sites. Nat Commun. 2021;12(1):4579. doi:10.1038/s41467-021-24861-8 34321471 PMC8319326

[10] World Cancer Research Fund/American Institute for Cancer Research . Continuous updated project expert Report 2018. Wholegrains, vegetables and fruit and the risk of cancer. https://www.wcrf.org/wp-content/uploads/2020/12/Wholegrains-veg-and-fruit.pdf

[11] Di Maso M , Bravi F , Polesel J , et al. Attributable fraction for multiple risk factors: methods, interpretations, and examples. Stat Methods Med Res. 2020;29(3):854‐865. doi:10.1177/0962280219848471 31074326

[12] Shield KD , Freisling H , Boutron‐Ruault MC , et al. New cancer cases attributable to diet among adults aged 30–84 years in France in 2015. Br J Nutr. 2018;120(10):1171‐1180. doi:10.1017/S0007114518002544 30401003

[13] Jiang X , Pestoni G , Vinci L , et al. Cancer cases attributable to modifiable lifestyle risk factors in Switzerland between 2015 and 2019. Int J Cancer. 2024;154(7):1221‐1234. doi:10.1002/ijc.34806 38041826

[14] Tybjerg AJ , Friis S , Brown K , Nilbert MC , Morch L , Koster B . Updated fraction of cancer attributable to lifestyle and environmental factors in Denmark in 2018. Sci Rep. 2022;12(1):549. doi:10.1038/s41598-021-04564-2 35017625 PMC8752838

[15] Parkin DM . 5. Cancers attributable to dietary factors in the UK in 2010. II. Meat consumption. Br J Cancer. 2011;105(Suppl 2):S24‐S26. doi:10.1038/bjc.2011.478 22158315 PMC3252055

[16] Parkin DM , Boyd L . 6. Cancers attributable to dietary factors in the UK in 2010. III. Low consumption of fibre. Br J Cancer. 2011;105(Suppl 2):S27‐S30. doi:10.1038/bjc.2011.479 22158316 PMC3252068

[17] Parkin DM , Boyd L . 4. Cancers attributable to dietary factors in the UK in 2010. I. Low consumption of fruit and vegetables. Br J Cancer. 2011;105(Suppl 2):S19‐S23. doi:10.1038/bjc.2011.477 22158313 PMC3252058

[18] Brown KF , Rumgay H , Dunlop C , et al. The fraction of cancer attributable to modifiable risk factors in England, Wales, Scotland, Northern Ireland, and the United Kingdom in 2015. Br J Cancer. 2018;118(8):1130‐1141. doi:10.1038/s41416-018-0029-6 29567982 PMC5931106

[19] Azevedo ESG , de Moura L , Curado MP , et al. The fraction of cancer attributable to ways of life, infections, occupation, and environmental agents in Brazil in 2020. PLoS One. 2016;11(2):e0148761. doi:10.1371/journal.pone.0148761 26863517 PMC4749327

[20] Islami F , Chen W , Yu XQ , et al. Cancer deaths and cases attributable to lifestyle factors and infections in China, 2013. Ann Oncol. 2017;28(10):2567‐2574. doi:10.1093/annonc/mdx342 28961829

[21] Gu MJ , Huang QC , Bao CZ , et al. Attributable causes of colorectal cancer in China. BMC Cancer. 2018;18(1):38. doi:10.1186/s12885-017-3968-z 29304763 PMC5756355

[22] Xiao HJ , Liang H , Wang JB , et al. Attributable causes of cancer in China: fruit and vegetable. Chin J Cancer Res. 2011;23(3):171‐176. doi:10.1007/s11670-011-0171-7 23467575 PMC3587560

[23] Chen W , Xia C , Zheng R , et al. Disparities by province, age, and sex in site‐specific cancer burden attributable to 23 potentially modifiable risk factors in China: a comparative risk assessment. Lancet Glob Health. 2019;7(2):e257‐e269. doi:10.1016/S2214-109X(18)30488-1 30683243

[24] Inoue M , Sawada N , Matsuda T , et al. Attributable causes of cancer in Japan in 2005–systematic assessment to estimate current burden of cancer attributable to known preventable risk factors in Japan. Ann Oncol. 2012;23(5):1362‐1369. doi:10.1093/annonc/mdr437 22048150

[25] Abe S , Takachi R , Ishihara J , et al. Burden of cancer attributable to excess red and processed meat consumption in Japan in 2015. GHM Open. 2021;1(2):91‐96. doi:10.35772/ghmo.2021.01019 PMC11933952 40145073

[26] Cho S , Shin A . Population attributable fraction of established modifiable risk factors on colorectal cancer in Korea. Cancer Res Treat. 2021;53(2):480‐486. doi:10.4143/crt.2019.742 33070559 PMC8053879

[27] Islami F , Goding Sauer A , Miller KD , et al. Proportion and number of cancer cases and deaths attributable to potentially modifiable risk factors in the United States. CA Cancer J Clin. 2018;68(1):31‐54. doi:10.3322/caac.21440 29160902

[28] Zhang FF , Cudhea F , Shan Z , et al. Preventable cancer burden associated with poor diet in the United States. JNCI Cancer Spectr. 2019;3(2):pkz034. doi:10.1093/jncics/pkz034 31360907 PMC6649723

[29] Grundy A , Poirier AE , Khandwala F , McFadden A , Friedenreich CM , Brenner DR . Cancer incidence attributable to red and processed meat consumption in Alberta in 2012. CMAJ Open. 2016;4(4):E768‐E775. doi:10.9778/cmajo.20160036 PMC5173478 28018893

[30] Poirier AE , Ruan Y , Hebert LA , et al. Estimates of the current and future burden of cancer attributable to low fruit and vegetable consumption in Canada. Prev Med. 2019;122:20‐30. doi:10.1016/j.ypmed.2019.03.013 31078169

[31] Ruan Y , Poirier AE , Hebert LA , et al. Estimates of the current and future burden of cancer attributable to red and processed meat consumption in Canada. Prev Med. 2019;122:31‐39. doi:10.1016/j.ypmed.2019.03.011 31078171

[32] Nagle CM , Wilson LF , Hughes MC , et al. Cancers in Australia in 2010 attributable to inadequate consumption of fruit, non‐starchy vegetables and dietary fibre. Aust N Z J Public Health. 2015;39(5):422‐428. doi:10.1111/1753-6405.12449 26437726 PMC4606769

[33] Nagle CM , Wilson LF , Hughes MC , et al. Cancers in Australia in 2010 attributable to the consumption of red and processed meat. Aust N Z J Public Health. 2015;39(5):429‐433. doi:10.1111/1753-6405.12450 26437727 PMC4606774

[34] Collatuzzo G , La Vecchia C , Parazzini F , et al. Cancers attributable to infectious agents in Italy. Eur J Cancer. 2023;183:69‐78. doi:10.1016/j.ejca.2023.01.010 36801622

[35] Collatuzzo G , Turati F , Malvezzi M , Negri E , La Vecchia C , Boffetta P . Attributable fraction of cancer related to occupational exposure in Italy. Cancer. 2023;15(8). 10.3390/cancers15082234 PMC10136839 37190163

[36] Di Maso M , Pelucchi C , Collatuzzo G , et al. Cancers attributable to overweight and obesity in Italy. Cancer Epidemiol. 2023;87:102468. doi:10.1016/j.canep.2023.102468 37832242

[37] Pizzato M , di Maso M , Collatuzzo G , et al. Cancer mortality associated with low education in Italy. J Public Health (Oxf). 2023;45(4):822‐828. doi:10.1093/pubmed/fdad164 37681283

[38] Collatuzzo G , Malvezzi M , Mangiaterra S , et al. Cancers attributable to tobacco smoking in Italy in 2020. Cancer Epidemiol. 2024;92:102623. doi:10.1016/j.canep.2024.102623 39018889

[39] World Cancer Research Fund/American Institute for Cancer Research . Continuous update project expert report 2018. Non‐alcoholic drinks and the risk of cancer. https://www.wcrf.org/wp-content/uploads/2021/02/Non-alcoholic-drinks.pdf

[40] Vieira AR , Abar L , Chan DSM , et al. Foods and beverages and colorectal cancer risk: a systematic review and meta‐analysis of cohort studies, an update of the evidence of the WCRF‐AICR continuous update project. Ann Oncol. 2017;28(8):1788‐1802. doi:10.1093/annonc/mdx171 28407090

[41] Vingeliene S , Chan DSM , Vieira AR , et al. An update of the WCRF/AICR systematic literature review and meta‐analysis on dietary and anthropometric factors and esophageal cancer risk. Ann Oncol. 2017;28(10):2409‐2419. doi:10.1093/annonc/mdx338 28666313 PMC5834025

[42] Vieira AR , Abar L , Vingeliene S , et al. Fruits, vegetables and lung cancer risk: a systematic review and meta‐analysis. Ann Oncol. 2016;27(1):81‐96. doi:10.1093/annonc/mdv381 26371287

[43] Maasland DH , van den Brandt PA , Kremer B , Goldbohm RA , Schouten LJ . Consumption of vegetables and fruits and risk of subtypes of head‐neck cancer in The Netherlands cohort study. Int J Cancer. 2015;136(5):E396‐E409. doi:10.1002/ijc.29219 25220255

[44] Vingeliene S , Chan DS , Aune D , et al. An update of the WCRF/AICR systematic literature review on esophageal and gastric cancers and citrus fruits intake. Cancer Causes Control. 2016;27(7):837‐851. doi:10.1007/s10552-016-0755-0 27153845 PMC4923099

[45] Bravi F , Tavani A , Bosetti C , Boffetta P , La Vecchia C . Coffee and the risk of hepatocellular carcinoma and chronic liver disease: a systematic review and meta‐analysis of prospective studies. Eur J Cancer Prev. 2017;26(5):368‐377. doi:10.1097/CEJ.0000000000000252 27111112

[46] Crous‐Bou M , Du M , Gunter MJ , et al. Coffee consumption and risk of endometrial cancer: a pooled analysis of individual participant data in the epidemiology of endometrial cancer consortium (E2C2). Am J Clin Nutr. 2022;116(5):1219‐1228. doi:10.1093/ajcn/nqac229 36041172 PMC9630862

[47] Turati F , Bravi F , Polesel J , et al. Adherence to the Mediterranean diet and nasopharyngeal cancer risk in Italy. Cancer Causes Control. 2017;28(2):89‐95. doi:10.1007/s10552-017-0850-x 28155006

[48] Leclercq C , Arcella D , Piccinelli R , et al. The Italian National Food Consumption Survey INRAN‐SCAI 2005‐06: main results in terms of food consumption. Public Health Nutr. 2009;12(12):2504‐2532. doi:10.1017/S1368980009005035 19278564

[49] Sette S , Le Donne C , Piccinelli R , et al. The third Italian National Food Consumption Survey, INRAN‐SCAI 2005‐06–part 1: nutrient intakes in Italy. Nutr Metab Cardiovasc Dis. 2011;21(12):922‐932. doi:10.1016/j.numecd.2010.03.001 20674305

[50] EFSA . Use of the EFSA comprehensive European food consumption database in exposure assessment. EFSA J. 2011;9(3):2097. doi:10.2903/j.efsa.2011.2097

[51] EFSA . Evaluation of the FoodEx, the food classification system applied to the development of the EFSA comprehensive European food consumption database. EFSA J. 2011;9(3):1970. doi:10.2903/j.efsa.2011.1970

[52] The concise encyclopedia of statistics . Version 1st. Springer. 2008.

[53] World Cancer Research Fund/American Institute for Cancer Research . Continuous update project expert report 2018. Recommendations and public health and policy implications. https://www.wcrf.org/wp-content/uploads/2021/01/Recommendations.pdf

[54] FAO and Ministry of Social Development and Family of Chile . Promoting safe and adequate fruit and vegetable consumption to improve health. Santiago de Chile. 2021 https://www.fao.org/3/cb7946en/cb7946en.pdf

[55] World Health Organization . WHO technical report series, No. 961. Diet, nutrition and the prevention of chronic diseases: report of a joint WHO/FAO expert consultation. 2003 https://iris.who.int/bitstream/handle/10665/42665/WHO_TRS_916.pdf 12768890

[56] Kalmpourtzidou A , Eilander A , Talsma EF . Global vegetable intake and supply compared to recommendations: a systematic review. Nutrients. 2020;12(6). doi:10.3390/nu12061558 PMC7352906 32471188

[57] Herforth A , Arimond M , Alvarez‐Sanchez C , Coates J , Christianson K , Muehlhoff E . A global review of food‐based dietary guidelines. Adv Nutr. 2019;10(4):590‐605. doi:10.1093/advances/nmy130 31041447 PMC6628851

[58] Levin ML . The occurrence of lung cancer in man. Acta Unio Int Contra Cancrum. 1953;9(3):531‐541. 13124110

[59] Mooney CZ , Sage P . Monte carlo simulation. Sage University Papers Series Quantitative Applications in the Social Sciences no 07–116. SAGE; 1997.

[60] Associazione Italiana di Oncologia Medica (AIOM) . I numeri del cancro in Italia 2019 . https://www.aiom.it/wp-content/uploads/2019/09/2019_Numeri_Cancro-operatori-web.pdf

[61] ISTAT . Indagine sui decessi e le cause di morte . http://dati.istat.it/

[62] Guasch‐Ferre M , Willett WC . The Mediterranean diet and health: a comprehensive overview. J Intern Med. 2021;290(3):549‐566. doi:10.1111/joim.13333 34423871

[63] Sanchez‐Sanchez ML , Garcia‐Vigara A , Hidalgo‐Mora JJ , Garcia‐Perez MA , Tarin J , Cano A . Mediterranean diet and health: a systematic review of epidemiological studies and intervention trials. Maturitas. 2020;136:25‐37. doi:10.1016/j.maturitas.2020.03.008 32386663

[64] Dinu M , Pagliai G , Casini A , Sofi F . Mediterranean diet and multiple health outcomes: an umbrella review of meta‐analyses of observational studies and randomised trials. Eur J Clin Nutr. 2018;72(1):30‐43. doi:10.1038/ejcn.2017.58 28488692

[65] Morze J , Danielewicz A , Przybylowicz K , Zeng H , Hoffmann G , Schwingshackl L . An updated systematic review and meta‐analysis on adherence to mediterranean diet and risk of cancer. Eur J Nutr. 2021;60(3):1561‐1586. doi:10.1007/s00394-020-02346-6 32770356 PMC7987633

[66] Kulhanova I , Znaor A , Shield KD , et al. Proportion of cancers attributable to major lifestyle and environmental risk factors in the eastern Mediterranean region. Int J Cancer. 2020;146(3):646‐656. doi:10.1002/ijc.32284 30882889

[67] Ishihara J , Takachi R , Abe S , et al. Burden of cancer attributable to insufficient vegetable, fruit and dietary fiber consumption in Japan in 2015. GHM Open. 2021;1(2):70‐75. doi:10.35772/ghmo.2021.01018 PMC11946562 40151562

[68] Poirier AE , Ruan Y , Hebert LA , et al. Corrigendum to “Estimates of the current and future burden of cancer attributable to low fruit and vegetable consumption in Canada” [Prev. Med. 122 (2019) 20–30]. Prev Med. 2019;125:79. doi:10.1016/j.ypmed.2019.05.013 31078169

[69] Di Maso M , Boffetta P , Negri E , La Vecchia C , Bravi F . Caffeinated coffee consumption and health outcomes in the US population: a dose‐response meta‐analysis and estimation of disease cases and deaths avoided. Adv Nutr. 2021;12(4):1160‐1176. doi:10.1093/advances/nmaa177 33570108 PMC8321867

[70] Zhou K , Lim T , Dodge JL , Terrault NA , Wilkens LR , Setiawan VW . Population‐attributable risk of modifiable lifestyle factors to hepatocellular carcinoma: the multi‐ethnic cohort. Aliment Pharmacol Ther. 2023;58(1):89‐98. doi:10.1111/apt.17523 37051717 PMC10810233

[71] Parish WJ , Aldridge A , Allaire B , et al. A new methodological approach to adjust alcohol exposure distributions to improve the estimation of alcohol‐attributable fractions. Addiction. 2017;112(11):2053‐2063. doi:10.1111/add.13880 28556274 PMC5854478

[72] Kehoe T , Gmel G , Shield KD , Gmel G , Rehm J . Determining the best population‐level alcohol consumption model and its impact on estimates of alcohol‐attributable harms. Popul Health Metr. 2012;10(6). doi:10.1186/1478-7954-10-6 PMC3352241 22490226

[73] Ferguson J , Alvarez A , Mulligan M , Judge C , O'Donnell M . Bias assessment and correction for Levin's population attributable fraction in the presence of confounding. Eur J Epidemiol. 2024;39(2):111‐119. doi:10.1007/s10654-023-01063-8 38170371 PMC10904572

[74] Ferlay J, Ervik M, Lam F , et al. Global cancer observatory: cancer today (version 1.1). Lyon, France: International Agency for Research on Cancer; 2024. Available from: https://gco.iarc.who.int/today. Accessed on 25, March 2024.

[75] O'Connell MM , Ferguson JP . Pathway‐specific population attributable fractions. Int J Epidemiol. 2022;51(6):1957‐1969. doi:10.1093/ije/dyac079 35536313 PMC9749703

