# Supplementary material for: Cancers attributable to diet in Italy
Source: Int J Cancer. 2024 Oct 24;156(6):1181–90. doi: 10.1002/ijc.35227 (PMC11737007; doi:10.1002/ijc.35227)
Supplement: Supplementary file 1 — Data S1. [file IJC-156-1181-s001.pdf]

Cancers attributable to diet in Italy

Federica Turati, Gianfranco Alicandro, Giulia Collatuzzo, Claudio Pelucchi, Matteo Malvezzi, Fabio Parazzini, Eva Negri, Paolo Boffetta, Carlo La Vecchia, Matteo Di Maso

Table of contents

Supplementary table 1 2

Supplementary table 2 3

Supplementary figure 1 5

Supplementary figure 2 6

References 7

Supplementary table 1. ICD-10 codes used to define site-specific cancer deaths and diagnoses in the national vital statistics on causes of death and Italian cancer registries databases, respectively.

Cancer site	ICD-10 code
Colon and rectum	C18-C20
Mouth, pharynx, and larynx	C00-C10; C12-C13; C14; C32
Nasopharynx	C11
Esophagus	C15
Lung	C34
Stomach	C16
Liver	C22
Endometrium	C54.1

Supplementary table 2. Population attributable fraction (PAF), observed and attributable cancer deaths in 2020, and corresponding 95% confidence interval (CI) for each dietary factor-cancer type pair by sex in Italy.

**Dietary factor/cancer type**	**Counterfactual distribution of dietary factor**[‡]	**Men**			**Women**		
		**PAF (95% CI), %**	**Observed deaths, n**	**Attributable deaths (95% CI), n**	**PAF (95% CI), %**	**Observed deaths, n**	**Attributable deaths (95% CI), n**
'Convincing' association*							
**Processed meat**	0 gr/day						
Colon and rectum [C18-C20]		10.5 (5.0-16.3)	10246	1076 (521-1670)	7.0 (3.4-11.0)	8716	610 (296-959)
'Probable' association*							
**Red meat**	Intake of <50 gr/day						
Colon and rectum [C18-C20]		3.3 (0.1-7.0)	10246	338 (10-717)	2.0 (0.1-4.2)	8716	174 (9-366)
**Dairy products**	Intake of ≥300 gr/day						
Colon and rectum [C18-C20]		4.8 (3.6-5.9)	10246	492 (369-605)	4.3 (3.2-5.3)	8716	375 (279-462)
**Fibre**	Intake of ≥30 gr/day						
Colon and rectum [C18-C20]		7.9 (0.4-14.5)	10246	809 (41-1486)	9.0 (0.5-16.8)	8716	784 (44-1464)
**Non-starchy vegetables**	Intake of ≥240 gr/day						
Mouth [C00-C10; C12-C13], pharynx [C14], and larynx [C32]		8.9 (0.0-18.1)	3160	281 (0-572)	9.7 (0.0-19.8)	1176	114 (0-233)
Nasopharynx [C11]		16.5 (5.2-26.8)	178[#]	29 (9-48)	17.8 (5.7-29.0)	73[#]	13 (4-21)
Esophagus, AC [C15]		6.3 (0.7-11.4)	447[†]	28 (3-51)	6.9 (0.7-12.6)	181[†]	12 (1-23)
Esophagus, SCC [C15]		5.1 (0.0-10.9)	907[†]	46 (0-99)	5.6 (0.0-12.1)	367[†]	21 (0-44)
Colon and rectum [C18-C20]		1.1 (0.3-1.8)	10246	113 (31-184)	1.2 (0.3-2.0)	8716	105 (26-174)
Lung [C34]		3.3 (0.8-5.5)	22188	732 (178-1220)	3.7 (0.9-6.2)	10110	374 (91-627)
**Fruit**	Intake of ≥160 gr/day						
Esophagus, SCC [C15]		7.3 (2.8-11.4)	907[†]	66 (25-103)	5.6 (2.1-8.8)	367[†]	21 (8-32)
Stomach [C16]		0.8 (0.0-2.3)	5082	41 (0-117)	0.6 (0.0-1.8)	3550	21 (0-64)
Lung [C34]		3.4 (2.3-4.5)	22188	754 (510-998)	2.6 (1.7-3.4)	10110	263 (172-344)
**Citrus fruit**	Any intake						
Stomach, cardia [C16.0]		6.3 (0.4-13.0)	762	48 (3-99)	7.1 (0.4-14.5)	533	38 (2-77)
**Coffee**	Any intake						
Liver [C22]		31.9 (19.7-42.8)	5708	1821 (1124-2443)	31.2 (19.2-42.0)	2882	899 (553-1210)
Endometrium [C54.1]		-	-	-	11.6 (4.1-19.0)	2152	250 (88-409)
**Diet-related cancers**[ⱡ]		13.9 (5.9-21.7)	47916	6674 (2815-10412)	13.9 (5.4-22.3)	29207	4074 (1573-6509)
**All cancers**[+]		6.8 (2.9-10.6)	97867	6674 (2815-10412)	5.1 (2.0-8.1)	79991	4074 (1573-6509)

[‡]Defined according to dietary intake recommendations of the World Cancer Research Fund/American Institute for Cancer Research (WCRF/AICR) or other Institutions [1-3]; *Defined according to the World Cancer Research Fund/American Institute for Cancer Research (WCRF/AICR) [4-8]; [#]Nasopharyngeal cancer deaths were estimated

as 8.7% and 6.9% of the total oral and pharyngeal cancer deaths in men (n=2050) and women (n=1061), respectively, according to a previous defined algorithm [9]; [†]Deaths from adenocarcinoma and squamous cell carcinoma of the esophagus were estimated as 33% and 67% of the total esophageal cancer deaths, respectively, according to a previous defined algorithm [9]; [ɫ]Diet-related cancer deaths included the following cancer sites: mouth, pharynx, larynx, nasopharynx, esophagus (adenocarcinoma and squamous cell carcinoma), stomach, colorectum, liver, lung, and endometrium; [+]All cancer deaths excluding non-melanoma skin cancer.

Supplementary figure 1. Estimated intake distributions for processed meat, red meat, dairy products, fibre, non-starchy vegetables, and fruit [10-13] in men according to gamma distribution [14]; observed proportions of no consumers and consumers for citrus fruit and coffee [10-13] in men.

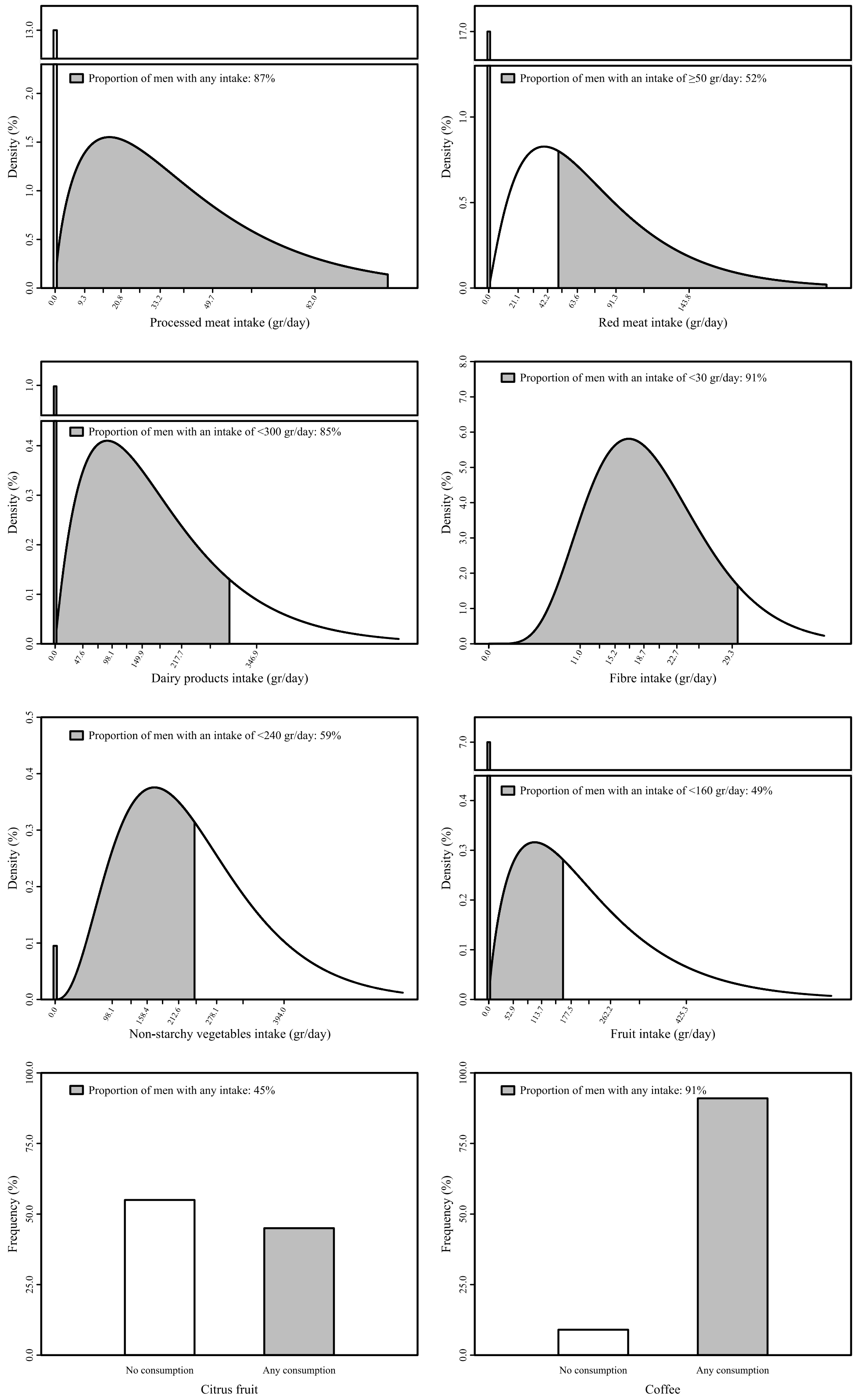

Supplementary figure 2. Estimated intake distributions for processed meat, red meat, dairy products, fibre, non-starchy vegetables, and fruit [10-13] in women according to gamma distribution [14]; observed proportions of no consumers and consumers for citrus fruit and coffee [10-13] in women.

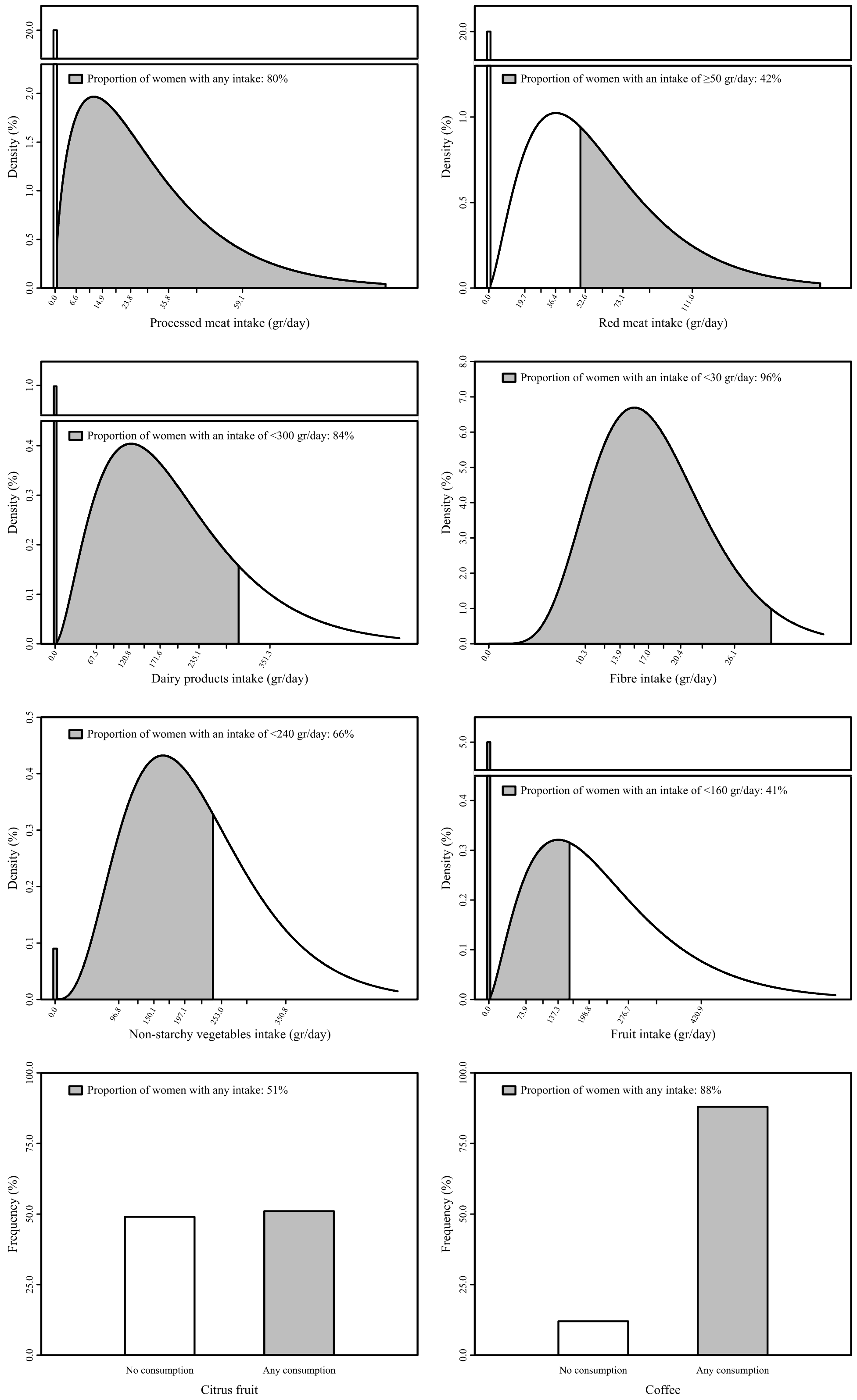

References

1. World Cancer Research Fund/American Institute for Cancer Research. Continuous Update Project Expert Report 2018. Recommendations and public health and policy implications. Available at: https://www.wcrf.org/wp-content/uploads/2021/01/Recommendations.pdf

2. FAO and Ministry of Social Development and Family of Chile. 2021. Promoting safe and adequate fruit and vegetable consumption to improve health. Santiago de Chile. Available at: https://www.fao.org/3/cb7946en/cb7946en.pdf

3. World Health Organization (2003) WHO Technical Report Series, No.961. Diet, nutrition and the prevention of chronic diseases: report of a Joint WHO/FAO Expert Consultation. Available at: https://iris.who.int/bitstream/handle/10665/42665/WHO_TRS_916.pdf

4. World Cancer Research Fund/American Institute for Cancer Research. Diet, Nutrition, Physical Activity and Cancer: a Global Perspective. Continuous Update Project Expert Report 2018. Available at: https://www.wcrf.org/wp-content/uploads/2021/02/Summary-of-Third-Expert-Report-2018.pdf

5. World Cancer Research Fund/American Institute for Cancer Research. Continuous Update Project Expert Report 2018. Diet, nutrition, physical activity and colorectal cancer. Available at: https://www.wcrf.org/wp-content/uploads/2021/02/Colorectal-cancer-report.pdf.

6. World Cancer Research Fund/American Institute for Cancer Research. Continuous Updated Project Expert Reporto 2018. Wholegrains, vegetabales and fruit and the risk of cancer. Available at: https://www.wcrf.org/wp-content/uploads/2020/12/Wholegrains-veg-and-fruit.pdf

7. World Cancer Research Fund/American Institute for Cancer Research. Continuous Update Project Expert Report 2018. Diet, nutrition, physical activity and stomach cancer. Available at: https://www.wcrf.org/wp-content/uploads/2021/02/stomach-cancer-report.pdf

8. World Cancer Research Fund/American Institute for Cancer Research. Continuous Update Project Expert Report 2018. Non-alcoholic drinks and the risk of cancer. Available at: https://www.wcrf.org/wp-content/uploads/2021/02/Non-alcoholic-drinks.pdf

9. Collatuzzo G, La Vecchia C, Parazzini F, Alicandro G, Turati F, Di Maso M, Malvezzi M, Pelucchi C, Negri E, Boffetta P. Cancers attributable to infectious agents in Italy. *Eur J Cancer*. Apr 2023;183:69-78. doi:10.1016/j.ejca.2023.01.010

10. Leclercq C, Arcella D, Piccinelli R, Sette S, Le Donne C, Turrini A, Group I-SS. The Italian National Food Consumption Survey INRAN-SCAI 2005-06: main results in terms of food consumption. *Public Health Nutr*. Dec 2009;12(12):2504-32. doi:10.1017/S1368980009005035

11. Sette S, Le Donne C, Piccinelli R, Arcella D, Turrini A, Leclercq C, Group I-SS. The third Italian National Food Consumption Survey, INRAN-SCAI 2005-06--part 1: nutrient intakes in Italy. *Nutr Metab Cardiovasc Dis*. Dec 2011;21(12):922-32. doi:10.1016/j.numecd.2010.03.001

12. EFSA, 2011. Use of the EFSA Comprehensive European Food Consumption Database in Exposure Assessment. EFSA J. 9(3), 2097. doi:10.2903/j.efsa.2011.2097

13. EFSA, 2011. Evaluation of the FoodEx, the food classification system applied to the development of the EFSA Comprehensive European Food Consumption Database. EFSA J. 9(3), 1970. doi:10.2903/j.efsa.2011.1970

14. *The concise encyclopedia of statistics*. Version 1st. Springer; 2008
